# Fermented Buckwheat Bee Pollen Attenuates LPS‐Induced Acute Inflammation in Mice by Suppressing Oxidative Stress and Inflammatory Response

**DOI:** 10.1155/ijfo/1609770

**Published:** 2026-05-27

**Authors:** Hua Wang, Tuya Naren, Cuifang Wang, Ayin Chen, Jinhua Liu, Wenxi Zhou, Shiying Tang

**Affiliations:** ^1^ College of Life Science and Food Engineering, Inner Mongolia Minzu University, Tongliao, Inner Mongolia, China, imun.edu.cn; ^2^ Tongliao Academy of Agricultural and Animal Husbandry Sciences, Tongliao, Inner Mongolia, China; ^3^ College of Food Science and Technology, Shanghai Ocean University, Shanghai, China, shou.edu.cn

**Keywords:** buckwheat bee pollen, enhanced bioactivity, fermentation, oxidative stress, systemic inflammation

## Abstract

This study is aimed at investigating the improvement effects of *Lactobacillus plantarum* fermentation on buckwheat bee pollen and its efficacy in alleviating lipopolysaccharide (LPS)‐induced acute inflammation. Through a combination of scanning electron microscopy observation, chemical composition analysis, in vitro antioxidant evaluation, and in vivo mouse experiments, the structural characteristics, active components, and functional properties of the fermented product were systematically investigated. The results showed that fermentation treatment significantly disrupted the sporopollenin exine of the pollen, leading to increases in total phenolic, total flavonoid, and free amino acid contents by 25.2%, 18.9%, and 49.5%, respectively. In vitro antioxidant assays demonstrated significant enhancements in ABTS, DPPH, and hydroxyl radical scavenging rates, as well as ferric ion reducing ability. In the LPS‐induced mouse acute inflammation model, the high‐dose fermented product (400 mg/kg) exhibited remarkable anti‐inflammatory effects, reducing TNF‐*α*, IL‐1*β*, and IL‐6 levels by 78.7%, 73.6%, and 33.4%, respectively, decreasing MPO activity by 59.8%, while significantly improving liver function indicators and enhancing hepatic antioxidant defense capacity. This study confirms that microbial fermentation can effectively enhance the bioactivity of buckwheat bee pollen, providing a theoretical basis and practical support for its development as a functional food ingredient.

## 1. Introduction

The development of functional ingredients from natural food resources represents a significant frontier in modern functional food science. With the global prevalence of metabolic diseases, growing scientific attention has focused on the pivotal role of oxidative stress and chronic low‐grade inflammation in disease pathogenesis. Substantial evidence indicates that persistent oxidative stress activates multiple inflammatory signaling pathways, creating a vicious cycle that ultimately leads to pathological alterations including insulin resistance and atherosclerosis [1–3]. This understanding has opened new avenues for dietary intervention strategies in preventing metabolic disorders.

Bioactive compounds derived from natural foods have garnered considerable interest due to their multitarget effects and favorable safety profiles. Plant‐derived polyphenols and flavonoids have demonstrated remarkable dual functionality in both antioxidant and anti‐inflammatory activities. Research has revealed that these natural compounds not only directly scavenge free radicals but also exert protective effects at the molecular level by modulating key signaling pathways such as Nrf2 and NF‐*κ*B [4]. For instance, dietary polyphenols can effectively mitigate oxidative stress by inhibiting NADPH oxidase activity and regulating antioxidant enzyme expression, while simultaneously suppressing proinflammatory factor production through interference with inflammasome activation [5].

Bee pollen, a natural nutrient repository collected by honeybees, is rich in proteins, vitamins, lipids, and bioactive compounds including polyphenols and flavonoids, earning its reputation as a “perfect complete nutritional food” [6]. Notably, buckwheat bee pollen contains significantly higher levels of polyphenolic compounds compared with other varieties, demonstrating superior in vitro antioxidant activity and considerable potential for development as a functional food ingredient [6, 7]. However, the rigid sporopollenin exine of bee pollen grains severely impedes the digestive absorption of its nutrients, creating a “bioavailability barrier” that substantially limits its high‐value utilization in the food industry [8, 9].

To overcome this limitation, bioprocessing techniques such as fermentation, germination, and enzymatic hydrolysis have been employed to modify raw material properties and enhance functional value. Microbial fermentation technology, characterized by mild conditions, high efficiency, and simultaneous flavor improvement, has been widely applied in functional food ingredient development. Indeed, similar fermentation strategies have proven successful in various natural matrices [10]. For example, lactic acid bacteria fermentation significantly increases soluble dietary fiber and phenolic content in cereals (e.g., oats and highland barley), enhancing their antioxidant activity, whereas *Aspergillus* or yeast fermentation is commonly used in legume materials (e.g., soybeans) to improve protein digestibility and generate novel bioactive components (e.g., soybean isoflavone aglycones) [11, 12]. The efficacy of these fermentations is largely attributed to the robust enzyme systems (e.g., cellulases, proteases, and *β*‐glucosidases) produced by microorganisms, which are capable of hydrolyzing complex plant cell wall polymers [13]. Given that the sporopollenin exine of pollen, while distinct, presents a similar accessibility challenge, these enzymatic actions are also hypothesized to disrupt the pollen wall structure and its adhesive pollenkitt layer [14].

Considerable evidence suggests that controlled fermentation can similarly disrupt the cellular wall structure of bee pollen, liberate encapsulated bioactive substances, and potentially generate new active components through microbial metabolic conversion, thereby synergistically enhancing its original biological activities [15, 16]. Nevertheless, although existing studies have confirmed that fermentation can improve the in vitro antioxidant capacity of bee pollen, the corresponding systemic anti‐inflammatory effects of fermentation‐enhanced bee pollen, particularly its regulatory impact on acute inflammation models, remain insufficiently explored, especially when compared with well‐established fermented materials. This knowledge gap constitutes a critical scientific barrier to its validation as a functional food ingredient.

Based on the research paradigm of employing food biotechnology to transform natural materials and enhance their health benefits, this study proposes the hypothesis that microbial fermentation can disrupt the physical barrier of buckwheat bee pollen, thereby releasing and transforming its functional components to significantly enhance its antioxidant and anti‐inflammatory activities. To systematically test this hypothesis, we first verified the structural and compositional improvements of pollen through scanning electron microscopy (SEM) and component analysis. Subsequently, we evaluated the enhancement of its activity through in vitro antioxidant assays. Finally, we comprehensively assessed its in vivo efficacy using an LPS‐induced mouse acute inflammation model, examining systemic inflammatory responses, liver function protection, and oxidative stress defense across three dimensions. This research is aimed at providing scientific evidence for developing fermented buckwheat bee pollen as a functional food ingredient with antioxidant and anti‐inflammatory properties, while offering new perspectives for adding value to natural food resources through bioprocessing technologies.

## 2. Materials and Methods

### 2.1. Materials

Buckwheat bee pollen was obtained from Lintao County, Gansu Province, China. The raw material was dried at 60°C to constant weight, ground using an FZ‐102 plant grinder (Tianjin Test Instrument Co. Ltd.), and sieved through an 80‐mesh screen. The resulting powder was aliquoted and stored at −80°C for subsequent use. *Lactobacillus plantarum* CICC 20279 was acquired from the China Center of Industrial Culture Collection. All chemical reagents were of analytical grade and used without further purification.

### 2.2. Fermentation Process of Buckwheat Bee Pollen

#### 2.2.1. Fermentation Procedure

*L. plantarum* was inoculated into MRS broth and cultured at 37°C for 24 h. Bacterial cells were harvested by centrifugation (3000 × g, 20 min), resuspended in sterile phosphate‐buffered saline (PBS, 1×, pH 6.8), and adjusted to a concentration of 10^8^ CFU/mL to serve as the inoculum. For substrate preparation, buckwheat bee pollen was mixed with sterile PBS at a 1:0.4 (w/w) ratio. The mixture was then subjected to thermal sterilization at 95°C for 15 min. It is important to note that this thermal treatment, applied to both the fermentation and control samples, may also serve as a pretreatment that induces initial swelling or microfractures in the pollen exine. After cooling to room temperature, the sterilized mixture for fermentation was inoculated with 10% (v/w) of the *L. plantarum* suspension and subjected to static fermentation at 37°C for 70 h. For the nonfermented control group, an equivalent portion of the sterilized pollen mixture was freeze‐dried immediately after cooling, without inoculation, and stored under identical conditions. Both the fermented and nonfermented control samples were subsequently freeze‐dried and stored at −20°C until further analysis.

#### 2.2.2. Composition Analysis

Standard methods were employed to determine the content of various components: total sugar content was measured by the phenol‐sulfuric acid method using glucose as the standard; protein content was determined via the Kjeldahl method with a conversion factor of 6.25; fat content was assessed by Soxhlet extraction using petroleum ether as the solvent; and ash content was determined by incineration at 550°C for 4 h. Total amino acids were analyzed using an amino acid analyzer after acid hydrolysis, whereas free amino acids were quantified by the ninhydrin colorimetric method with leucine as the standard. Total flavonoid content was determined by the aluminum nitrate colorimetric method using rutin as the standard, and total phenolic content was measured using the Folin–Ciocalteu method with gallic acid as the standard. All assays were performed in triplicate, and results were expressed on a dry weight basis.

#### 2.2.3. SEM

The microscopic structure of the samples was examined using a Hitachi SU8010 scanning electron microscope. Samples were evenly dispersed on conductive adhesive, sputter‐coated with gold under vacuum, and observed at accelerating voltages of 5.0 kV. Images were captured at magnifications of 500×, 2000×, and 5000× to compare surface structural changes of pollen particles before and after fermentation.

### 2.3. In Vitro Antioxidant Activity Assays

All in vitro antioxidant activity assays were performed using supernatant obtained from 60% ethanol extraction. Specifically, 0.05 g of sample was accurately weighed, mixed with 1.00 mL of 60% ethanol solution, and extracted in a 40°C water bath for 30 min. The mixture was then centrifuged at 10,000 rpm for 10 min at 25°C, and the supernatant was collected as the test solution.

#### 2.3.1. ABTS Radical Scavenging Activity

The assay was conducted following the method of Mehta et al. with minor modifications [17]. The ABTS stock solution was prepared by mixing 7‐mmol/L ABTS solution with 2.45‐mmol/L potassium persulfate solution in equal volumes, followed by incubation in the dark at room temperature for 12 h. Before measurement, the stock solution was diluted with phosphate buffer (pH 7.4) to an absorbance of 0.70 ± 0.02 at 734 nm. Then, 0.1 mL of the sample supernatant was mixed with 2.9 mL of the diluted working solution, reacted at room temperature for 6 min, and the absorbance at 734 nm was immediately measured. The ABTS radical scavenging rate was calculated using Equation (1).

(1) ABTS radical scavenging capacity %=A734 blank−A734 sampleA734 sample×100

#### 2.3.2. DPPH Radical Scavenging Activity

The method described by Brand‐Williams et al. was adopted [18]. A total of 2 mL of sample supernatant was mixed with 2 mL of 0.2‐mmol/L DPPH ethanol solution and incubated in the dark at room temperature for 30 min. The absorbance was measured at 517 nm. An equal volume of anhydrous ethanol was used to replace the DPPH solution as the sample background control, and an equal volume of extraction solvent served as the blank control. The DPPH radical scavenging rate was calculated using Equation (2).

(2) DPPH radical scavenging capacity %=1−A517 sampleA517 blank×100

#### 2.3.3. Hydroxyl Radical Scavenging Activity

The hydroxyl radical scavenging capacity was evaluated using the salicylic acid capture method. A total of 1 mL of sample supernatant, 1 mL of 9‐mmol/L FeSO_4_ solution, 1 mL of 9‐mmol/L salicylic acid–ethanol solution, and 1 mL of 8.8‐mmol/L H_2_O_2_ solution were sequentially added. The mixture was incubated in a 37°C water bath for 30 min, and the absorbance was measured at 510 nm. Distilled water was used to replace the sample supernatant as the blank control, and distilled water was used instead of the salicylic acid–ethanol solution as the sample background control. The hydroxyl radical scavenging rate was calculated using Equation (3).

(3) Hydroxyl radical scavenging capacity %=1−A510 sample−A510controlA510 blank×100

#### 2.3.4. Ferric Ion Reducing Antioxidant Power (FRAP)

The FRAP assay was performed according to the method of Benzie and Strain [19]. The FRAP working solution was freshly prepared by mixing 0.3‐mol/L acetate buffer (pH 3.6), 10‐mmol/L TPTZ solution (dissolved in 40 mmol/L HCl), and 20‐mmol/L FeCl_3_ solution in a 10:1:1 (v/v/v) ratio. Then, 0.1 mL of sample supernatant was combined with 3 mL of FRAP working solution, incubated at 37°C in the dark for 10 min, and the absorbance was measured at 593 nm. A standard curve was constructed using ferrous sulfate solutions of varying concentrations. The absorbance values of the samples were then substituted into the standard curve equation to determine the Fe^2+^ equivalent concentration, and the results were expressed as *μ*mol Fe^2+^ equivalent per gram of sample.

#### 2.3.5. Hyaluronidase Inhibitory Activity

The hyaluronidase inhibition assay was employed to evaluate in vitro anti‐inflammatory activity. The sample solution was preincubated with hyaluronidase solution at 37°C for 10 min, after which sodium hyaluronate was added to initiate the reaction. The mixture was incubated at 37°C for 40 min, followed by the sequential addition of sodium hydroxide and acetylacetone solutions. After heating in a boiling water bath, p‐dimethylaminobenzaldehyde chromogenic agent was added, and the absorbance was measured at 530 nm to calculate the inhibition rate. The hyaluronidase inhibition activity was calculated using Equation (4).

(4) Hyaluronidase inhibition activity %=1−A530 sample−A530 sample backgroundA530 enzyme control−A530 reagent blank×100

### 2.4. Animal Experiment Design

A total of 60 specific pathogen‐free (SPF) male C57BL/6J mice (SCXK [Jing] 2020‐0008) were purchased from Sipeifu (Beijing) Biotechnology Co. Ltd. All animal experimental procedures were approved by the Animal Ethics Committee of Inner Mongolia Minzu University (Approval No.: NMD‐DW‐2025‐03‐50) and conducted in accordance with the ARRIVE guidelines. Mice were housed in an individually ventilated caging (IVC) system under controlled conditions: temperature 23^°^C ± 2^°^C, humidity 50*%* ± 10*%*, and a 12/12 h light/dark cycle, with ad libitum access to food and water. After 1 week of acclimatization, the mice were randomly divided into four groups (*n* = 15 per group) based on body weight: the control group (Con), the LPS model group (Model), the low‐dose fermented bee pollen group (BBFP‐L), and the high‐dose fermented bee pollen group (BBFP‐H). For oral administration, the freeze‐dried fermented buckwheat bee pollen powder was resuspended in physiological saline to achieve concentrations of 10 and 40 mg/mL. Mice in the BBFP‐L and BBFP‐H groups were then gavaged with 0.2 mL of this suspension, corresponding to doses of 100 and 400 mg/kg body weight, respectively. The dosage levels were selected based on a human equivalent dose conversion following the method of Reagan‐Shaw et al. [20]. The Con and the LPS model group received an equal volume of physiological saline by gavage. After 15 days of continuous intervention, except for the control group, all other groups were intraperitoneally injected with 10 mg/kg of lipopolysaccharide (LPS) to establish an acute inflammation model. At 4 h postinjection, blood and tissue samples were collected. Mice were deeply anesthetized with an intraperitoneal injection of pentobarbital sodium (50 mg/kg), and blood was collected from the retro‐orbital plexus. Subsequently, mice were euthanized by cervical dislocation to ensure minimal distress. Liver and spleen were excised and weighed to calculate organ indices. Liver tissues were flash‐frozen in liquid nitrogen and stored at −80°C, and serum was separated by centrifugation and stored at −80°C for subsequent analyses.

### 2.5. Biochemical Analysis

Serum levels of TNF‐*α*, IL‐1*β*, and IL‐6 were detected using enzyme‐linked immunosorbent assay (ELISA) kits (BioLegend). Commercial kits (Nanjing Jiancheng Bioengineering Institute) were used to measure serum ALT, AST, and MPO activities, as well as hepatic MDA content, CAT, GSH‐PX, T‐SOD activities, and T‐AOC levels. All procedures were strictly performed according to the manufacturers′ instructions.

### 2.6. Statistical Analysis

Data were processed using GraphPad Prism 9 software (GraphPad Software, San Diego, California, United States) and expressed as mean ± standard deviation (SD). Normal distribution was assessed using the Shapiro–Wilk test. One‐way analysis of variance (ANOVA) followed by Tukey′s post hoc test was employed to assess differences among groups. A *p* value of < 0.05 was considered statistically significant. All experiments were performed with *n* = 8 independent replicates.

## 3. Results

### 3.1. Effect of Fermentation on the Composition and Microstructure of Buckwheat Bee Pollen

**Figure 1:**
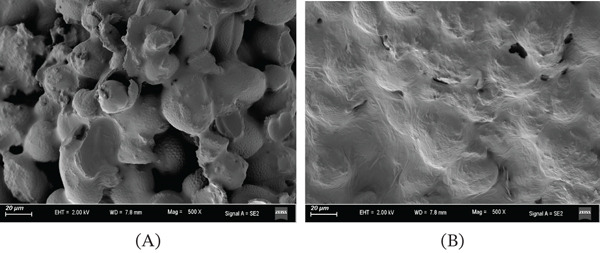
Microstructural changes of buckwheat bee pollen before and after fermentation (500×). (A) Unfermented buckwheat bee pollen. (B) Fermented buckwheat bee pollen.

Microbial fermentation serves as an effective bioprocessing strategy, the core value of which lies in reshaping the composition of raw materials and enhancing their functional properties through microbial metabolism [21]. In this study, SEM observations revealed that *L. plantarum* fermentation induced fundamental alterations in the microstructure of buckwheat bee pollen (Figure 1). Unfermented pollen grains exhibited intact morphology with smooth and dense surfaces, attributable to their natural sporopollenin exine, which acts as the primary physical barrier limiting the release of internal components. In contrast, fermented pollen grains underwent significant morphological changes: surfaces became rough, showing apparent shrinkage, collapse, and pore formation, with some particles even rupturing completely and releasing their contents. These visual morphological evidences demonstrate that the fermentation process effectively disrupted the pollen cell wall structure. Compositional analysis results indicated that fermentation significantly altered the chemical profile of buckwheat bee pollen (Table 1). The enhancement of bioactive components was particularly notable: total phenolic and total flavonoid contents increased by 25.2% and 18.9%, respectively. This marked increase is primarily attributed to two concurrent processes: the enzymatic system produced by *L. plantarum* effectively degraded the sporopollenin exine, breaking the physical barrier and facilitating the release of encapsulated phenolic and flavonoid compounds; simultaneously, microbial metabolism potentially converted some bound polyphenols into free forms, further augmenting the content of active components [22, 23].

**Table 1:** Changes in the bioactivity and nutritional composition of buckwheat bee pollen after fermentation.

Bioactivity/nutritional composition parameter	Unfermented buckwheat bee pollen	Fermented buckwheat bee pollen
Total phenol content (mg GAE/g)	3.02 ± 0.01^b^	3.78 ± 0.08^a^
Total flavonoid content (mg CE/g)	2.91 ± 0.01^b^	3.46 ± 0.01^a^
Total amino acid (*μ*mol/g)	28.30 ± 0.21^b^	31.28 ± 0.43^a^
Free amino acid (mg/g)	5.27 ± 0.03^b^	7.88 ± 0.01^a^
Total protein (%)	16.91 ± 0.05^a^	14.34 ± 0.71^b^
Total sugar (%)	36.71 ± 0.01^a^	33.45 ± 1.09^b^
Fat (%)	3.76 ± 0.23^b^	4.68 ± 0.07^a^
Ash (%)	4.58 ± 0.01^b^	4.74 ± 0.11^a^

*Note:* Data are presented as mean ± SD. Different superscript letters (a, b) within the same row indicate statistically significant differences at *p* < 0.05.

Abbreviations: CE, catechin equivalents; GAE, gallic acid equivalents.

These findings align with recent studies reporting the enhancement of bioactivity in plant matrices through microbial fermentation. Regarding nutritional components, fermentation induced significant redistribution: total amino acid content increased by 10.5%, whereas free amino acid content surged substantially by 49.5%, accompanied by a 15.2% decrease in total protein content. This trend clearly demonstrates the proteolytic action of microbial fermentation, converting macromolecular proteins into more readily absorbable small peptides and amino acids. This “predigestion” effect not only improves the product′s nutritional value but also, more importantly, generates a substantial amount of physiologically active small molecules that may significantly enhance its bioavailability [24, 25]. Furthermore, basic composition analysis revealed an 8.9% decrease in total sugar content during fermentation, providing direct evidence of microbial growth and metabolism, indicating that *L. plantarum* utilized pollen sugars as the primary carbon source. The 24.5% increase in fat content and slight elevation in ash content may be associated with microbial biosynthesis and transformation activities [26]. Collectively, these systematic compositional changes demonstrate that the fermentation process not only disrupted physical barriers but also reshaped the pollen′s chemical profile through biotransformation. The significant enhancement of key bioactive components, particularly the near‐doubling of free amino acids, establishes a solid material foundation for the improvement of its functional activity.

### 3.2. In Vitro Antioxidant Activity Evaluation of Fermented Buckwheat Bee Pollen

This study systematically analyzed the effect of fermentation on the antioxidant capacity of buckwheat bee pollen using four complementary in vitro antioxidant evaluation systems. The results (Table 2) demonstrated that *L. plantarum* fermentation significantly enhanced the comprehensive antioxidant capacity of the samples. Regarding free radical scavenging ability, the fermented product exhibited comprehensively enhanced characteristics. The ABTS^+^ radical scavenging rate increased by approximately 20%, indicating enhanced ability to scavenge hydrophilic radicals; the DPPH radical scavenging rate improved by about 14%, demonstrating improved quenching capacity for lipophilic radicals. Particularly noteworthy, the hydroxyl radical scavenging activity of the fermented product increased by approximately 30%, a finding of significant biological importance as the hydroxyl radical is the most reactive oxygen radical in biological systems. At the antioxidant mechanism level, the FRAP assay results provided deeper insights. The ferric ion reducing power of the fermented sample increased by over 50%, confirming not only a significantly enhanced ability to terminate free radical chain reactions via electron transfer but also revealing the diversity in the antioxidant mechanisms of the fermented product [27].

**Table 2:** Changes in the in vitro bioactivity of buckwheat bee pollen after fermentation.

In vitro bioactivity assay	Unfermented buckwheat bee pollen	Fermented buckwheat bee pollen
ABTS radical scavenging activity (%)	43.74 ± 0.16^b^	52.60 ± 1.75^a^
DPPH radical scavenging activity (%)	64.50 ± 0.05^b^	73.69 ± 0.18^a^
Hydroxyl radical scavenging activity (%)	54.60 ± 1.15^b^	71.09 ± 1.13^a^
FRAP (*μ*mol/g)	14.17 ± 0.10^b^	21.37 ± 0.26^a^
Hyaluronidase inhibitory activity (%)	9.13 ± 0.22^b^	25.79 ± 0.80^a^

*Note:* Data are presented as mean ± standard deviation (SD). Different superscript letters (a, b) within the same row indicate statistically significant differences at *p* < 0.05.

Analysis of the component–activity relationship indicated a significant correlation between the overall enhancement of antioxidant capacity and the increased contents of total phenolics and flavonoids. The phenolic and flavonoid compounds released during fermentation not only directly participate in radical neutralization reactions, but also undergo structural modifications that may enhance their antioxidant efficacy [28]. Additionally, the substantial increase in free‐amino acid content potentially contributes to the enhanced antioxidant activity through multiple pathways: on one hand, certain amino acids possess inherent radical scavenging capabilities; on the other hand, amino acids may form synergistic interactions with polyphenols, enhancing their stability and activity [29]. The differential results across various antioxidant evaluation systems reflect the complexity of the antioxidant properties of the fermented product. The outstanding performance observed in the FRAP system suggests that fermentation may preferentially enhance the electron‐donating capacity of the sample, while its excellent performance in hydroxyl radical scavenging reflects its special advantage against highly reactive oxygen species (ROS) [30]. These characteristics render it more widely applicable in countering different types of oxidative stress. Compared with recent studies, the magnitude of antioxidant activity enhancement observed in this study significantly exceeds those reported for conventional plant matrices subjected to fermentation. This may be related to the unique composition of bee pollen and the special structure of its sporopollenin exine. *L. plantarum* fermentation not only disrupted the physical barrier of bee pollen but also, more importantly, optimized the composition and conformation of active components through biotransformation, thereby achieving a remarkable enhancement in antioxidant activity.

### 3.3. Protective Effects of Fermented Buckwheat Bee Pollen Against LPS‐Induced Inflammation

#### 3.3.1. Fermented Product Alleviates Systemic Inflammatory Response

**Figure 2:**
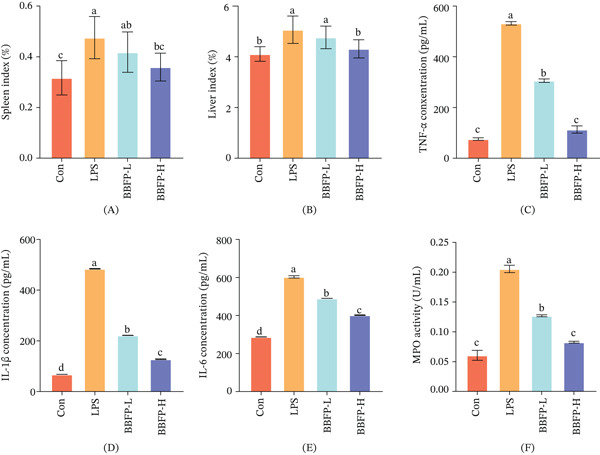
Effects of fermented buckwheat bee pollen on organ index and oxidative stress in LPS‐induced mice. (A) Spleen index, (B) liver index, (C) TNF‐*α* concentration, (D) IL‐1*β* concentration, (E) IL‐6 concentration, and (F) MPO activity. Control, normal control group; LPS, LPS‐induced model group; BBFP‐L, buckwheat bee pollen product low‐dose group; BBFP‐H, buckwheat bee pollen product high‐dose group. Different lowercase superscript letters within the same row indicate significant differences (*p* < 0.05).

This study systematically evaluated the regulatory effects of fermented buckwheat bee pollen on the systemic inflammatory response induced by LPS using organ indices and serum inflammatory cytokines, among other indicators. Organ index analysis revealed that LPS challenge significantly increased the spleen index and liver index by 50% and 23%, respectively (*p* < 0.05) (Figure 2A,B). This phenomenon reflects typical pathological alterations in the acute inflammatory state, including congestion and enlargement of immune organs and massive infiltration of inflammatory cells. Notably, intervention with the fermented buckwheat bee pollen significantly ameliorated these pathological changes. The low‐dose group (BBFP‐L) showed reductions in spleen index and liver index by 12.5% and 5.9%, respectively, compared with the model group, whereas the high‐dose group (BBFP‐H) exhibited more pronounced effects, with reductions of 25% and 15%, respectively, nearly restoring them to normal levels. These results demonstrate at a macroscopic level that the fermented product effectively alleviates the LPS‐induced systemic inflammatory response and associated organ pathology.

At the molecular level, our analysis of key serum inflammatory cytokines further revealed the anti‐inflammatory properties of the fermented product (Figure 2C–E). LPS stimulation triggered a typical “cytokine storm,” with serum levels of IL‐1*β*, IL‐6, and TNF‐*α* in the model group significantly elevated by 7.1‐, 2.1‐, and 7.0‐fold, respectively (*p* < 0.05), compared with the control group. Intervention with the fermented product significantly suppressed this inflammatory cascade in a clear dose‐dependent manner. Particularly noteworthy, the high‐dose group showed the most prominent inhibition of TNF‐*α*, with a reduction of 78.7%, whereas IL‐1*β* and IL‐6 decreased by 73.6% and 33.4%, respectively. This finding holds significant physiological importance because TNF‐*α*, as an early key mediator of the inflammatory response, plays a central role in initiating and amplifying the inflammatory cascade [31]. This significant anti‐inflammatory effect is closely related to the chemical changes in the fermented product. As mentioned previously, the contents of total phenolics and total flavonoids were significantly increased by 25.2% and 18.9%, respectively, after fermentation [5]. Numerous studies have indicated that dietary polyphenols can mitigate oxidative stress through their potent antioxidant activity, thereby inhibiting the expression of inflammatory factors. For instance, Lauren et al. [32] found that apple polyphenol extract could inhibit TNF‐*α* production in LPS‐induced macrophages by scavenging free radicals. Our results are consistent with this, suggesting that the enriched polyphenols and flavonoids in fermented buckwheat bee pollen likely constitute the important material basis for its anti‐inflammatory effects.

Furthermore, changes in MPO activity provided direct evidence for neutrophil infiltration (Figure 2F). MPO activity in the model group increased to 3.4 times that of the control group, whereas the low‐ and high‐dose intervention groups reduced it by 38.2% and 59.8%, respectively. This result suggests that the fermented product may alleviate tissue inflammatory damage by inhibiting the activation and migration of neutrophils. It is worth noting that the significantly increased flavonoid components in the fermented product likely play an important role in this process. Studies have shown that flavonoids such as rutin and quercetin can inhibit the adhesion of neutrophils to vascular endothelial cells, thereby reducing neutrophil recruitment to inflammatory sites [33]. This aligns with our findings and further supports the notion that the fermented product exerts its anti‐inflammatory effects through multiple pathways.

#### 3.3.2. Fermented Product Ameliorates Liver Function Injury

**Figure 3:**
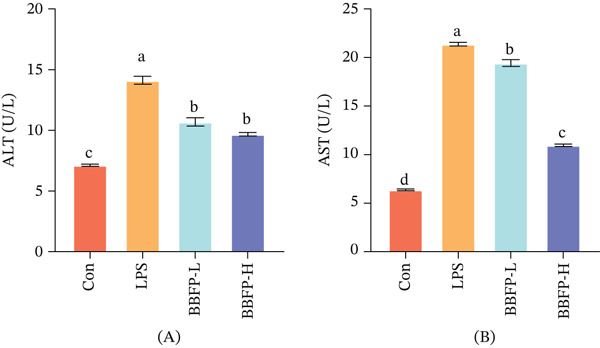
Effects of fermented buckwheat bee pollen on ALT and AST stress in LPS‐induced mice. (A) ALT and (B) AST. Control, normal control group; LPS, LPS‐induced model group; BBFP‐L, buckwheat bee pollen product low‐dose group; BBFP‐H, buckwheat bee pollen product high‐dose group. Different lowercase superscript letters within the same row indicate significant differences (*p* < 0.05).

The liver, as the metabolic center and primary detoxification organ of the body, is highly vulnerable to damage during systemic inflammatory responses. This study evaluated the hepatoprotective effects of the fermented product by measuring serum transaminase levels (Figure 3). The results showed that LPS challenge significantly increased ALT and AST activities by 98.0% and 235.5%, respectively, indicating severe disruption of hepatocyte membrane integrity and potential mitochondrial damage. The significant increase in the AST/ALT ratio (from 0.89 in the control group to 1.51 in the model group) further supports this speculation, as AST is primarily located in mitochondria, and its substantial release often suggests more severe organelle damage [34].

Intervention with the fermented product significantly improved liver function status, showing a dose‐dependent protective effect. The low‐dose group reduced ALT and AST levels by 24.3% and 9.1%, respectively, compared with the model group, whereas the high‐dose group showed more significant effects, with reductions of 31.4% and 48.8%, respectively. Particularly noteworthy, the high‐dose group′s effect on reducing AST was markedly superior to that on ALT, with the AST/ALT ratio recovering to 1.13. This may suggest a special protective effect of the fermented product on mitochondrial function. This finding is closely related to the significantly increased free amino acid composition in the fermented product. Research indicates that certain amino acids and their metabolites play important roles in maintaining cellular energy metabolism and mitochondrial function. For example, glutamate and aspartate participate in the tricarboxylic acid cycle, whereas sulfur‐containing amino acids such as cysteine are precursors for glutathione synthesis; these amino acids may exert protective effects by maintaining mitochondrial metabolic homeostasis [35].

Analyzing the protective mechanisms, the hepatoprotective effect of the fermented product may stem from the synergistic action of multiple aspects. Firstly, by inhibiting the systemic inflammatory response, it reduces the direct attack of inflammatory mediators on hepatocytes. Secondly, the bioactive components produced during fermentation may directly act on hepatocytes, stabilizing cell membrane structure. Furthermore, its excellent antioxidant activity also helps maintain the functional integrity of hepatocytes. The concerted action of these multiple protective mechanisms ultimately achieves comprehensive protection of liver function.

#### 3.3.3. Fermented Product Enhances Hepatic Antioxidant Defense Capacity

**Figure 4:**
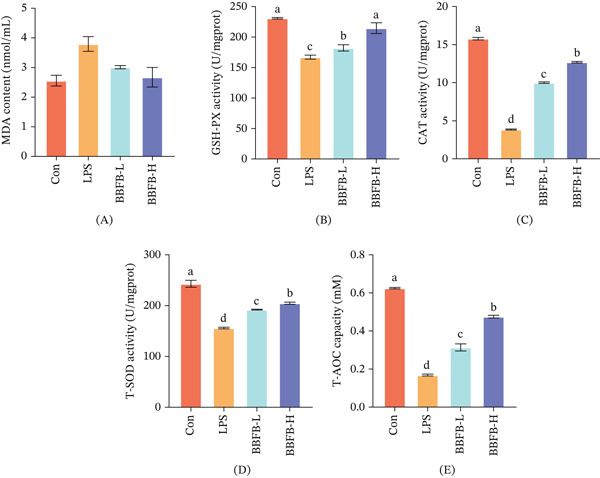
Effects of fermented buckwheat bee pollen on oxidative stress markers in LPS‐induced mice. (A) MDA content, (B) GSH‐PX activity, (C) CAT activity, (D) T‐SOD activity, and (E) T‐AOC capacity. Control, normal control group; LPS, LPS‐induced model group; BBFP‐L, buckwheat bee pollen product low‐dose group; BBFP‐H, buckwheat bee pollen product high‐dose group. Different lowercase superscript letters within the same row indicate significant differences (*p* < 0.05).

To further investigate the hepatoprotective mechanisms of fermented buckwheat bee pollen, we systematically assessed the oxidative stress status in liver tissue. The results showed that LPS challenge not only promoted ROS production by activating NADPH oxidase but also simultaneously suppressed the endogenous antioxidant defense system, leading to a significant 40.2% increase in the lipid peroxidation product MDA content in the liver, whereas GSH‐PX, CAT, T‐SOD activities, and T‐AOC decreased by 27.7%, 75.6%, 35.8%, and 73.0%, respectively (Figure 4). This severe imbalance between oxidation and antioxidation is an important mechanism leading to hepatocyte damage. Intervention with the fermented product effectively reversed this pathological state. The low‐dose group already showed a clear improving trend, whereas the high‐dose group was particularly effective: MDA levels returned to the normal range (Figure 4A), whereas GSH‐PX, CAT, T‐SOD activities, and T‐AOC were restored to 92.9%, 80.4%, 84.3%, and 65.5% of the control group levels, respectively (Figure 4B–E). Notably, the degree of recovery varied among different antioxidant indicators, with CAT activity showing the most significant improvement, recovering from a mere 24.4% to 80.4% (Figure 4C). This phenomenon holds significant biological importance because CAT is a key enzyme for scavenging hydrogen peroxide, and its effective recovery is crucial for preventing the formation of •OH radicals [36, 37]. The •OH radical is known as the most reactive oxygen radical, capable of reacting with almost all biological molecules, causing severe cellular damage.

This exceptional antioxidant effect is closely related to the chemical composition characteristics of the fermented product. Firstly, the substantial increases in total phenolic and total flavonoid contents (by 25.2% and 18.9%, respectively) directly enhanced the free radical scavenging capacity of the system. Numerous studies have shown that phenolic compounds can not only directly quench free radicals but may also exert protective effects by regulating the expression of the antioxidant defense system. For instance, certain polyphenolic components have been confirmed to activate the Nrf2‐ARE signaling pathway, thereby upregulating the expression of various antioxidant enzymes [38]. Secondly, the significant 49.5% increase in free‐amino acid content also holds important functional significance: sulfur‐containing amino acids (e.g., cysteine) are precursors for glutathione synthesis, whereas amino acids such as histidine and proline themselves possess metal ion chelating ability, which can inhibit •OH radical generation via the Fenton reaction [39]. The synergistic effects of these components collectively constitute the powerful antioxidant defense network of the fermented product.

Therefore, this study systematically reveals the multiple mechanisms by which microbial fermentation technology enhances the bioactivity of buckwheat bee pollen. The fermentation process not only promoted the release of active components by disrupting the physical barrier of the sporopollenin exine but also, more importantly, reshaped its chemical profile through biotransformation. The significant increase in total phenolic and total flavonoid contents directly enhanced the free radical scavenging capacity of the system, whereas the substantial increase in free amino acids contributed to the overall bioactivity through multiple pathways: acting as antioxidants to directly participate in free radical quenching on one hand, and serving as important metabolic precursors involved in the construction of intracellular antioxidant defense systems on the other. This synergistic effect of components is the material basis for the exceptional functional activity exhibited by the fermented product. At the mechanistic level, fermented buckwheat bee pollen exhibits multitarget and multilevel characteristics. Its alleviating effect on LPS‐induced inflammation is manifested not only by the inhibition of proinflammatory factor production at the systemic level and the restriction of neutrophil infiltration at the cellular level but also extends to the enhancement of the hepatocyte antioxidant defense system at the molecular level. Particularly noteworthy is that the high‐dose group (400 mg/kg) showed optimal effects across all evaluation indicators, providing an important dosage reference for subsequent application studies. The improving effect of the fermented product on the AST/ALT ratio suggests its potential special protective value for mitochondrial function, a discovery that provides a new direction for in‐depth exploration of its mechanisms of action.

Although this study has yielded valuable findings, several issues require further investigation. For example, the transformation patterns of specific active components during fermentation and their structure–activity relationships with functional activity need further elucidation; the metabolic fate and target sites of the fermented product in vivo remain to be clarified; personalized dosage regimens based on different health needs still require optimization. Addressing these issues will help promote the transition of fermented buckwheat bee pollen from experimental research to practical application, providing new impetus for the development of the functional food industry.

## 4. Conclusion

This study systematically elucidates the enhancement effects of *L. plantarum* fermentation on buckwheat bee pollen, spanning from structural characteristics and chemical composition to functional activity. The fermentation process not only disrupts the physical barrier of pollen to promote the release of active components but also optimizes its chemical profile through biotransformation. These changes constitute the material basis for the enhanced functionality of the fermented product. In vitro experiments demonstrated that fermentation significantly strengthened the antioxidant capacity of the product. In vivo studies further revealed its multitarget and multilevel mechanisms in alleviating LPS‐induced acute inflammation: at the systemic level by inhibiting inflammatory cytokine production and neutrophil infiltration; at the organ level by protecting liver function, with particular effects on mitochondria; and at the molecular level by enhancing the endogenous antioxidant defense system. Notably, the high‐dose fermented product (400 mg/kg) exhibited optimal effects across all evaluation indicators, providing a crucial reference for practical application. In conclusion, this study not only provides an innovative approach for the value‐added utilization of buckwheat bee pollen but also establishes a solid foundation for developing fermentation‐based functional food ingredients, showing promising application prospects in the functional food sector.

## Author Contributions

Hua Wang: conceptualization, methodology, and funding acquisition. Tuya Naren: writing—original draft preparation, software, and data curation. Cuifang Wang: validation. Ayin Chen: formal analysis. Jinhua Liu: resources. Wenxi Zhou: data curation and project administration. Shiying Tang: writing—review and editing.

## Funding

This study was supported by Natural Science Foundation of Inner Mongolia Autonomous Region of China (2024MS03060); Inner Mongolia Youth Science Fund (2021BS0824); Innovation and Entrepreneurship Training Program for College Students of Inner Mongolia Autonomous Region (S202510136025).

## Disclosure

All authors have read and agreed to the published version of the manuscript.

## Conflicts of Interest

The authors declare no conflicts of interest.

## Data Availability

The data supporting the findings of this study are available on request from the corresponding authors.
